# LRP5: A Multifaceted Co-Receptor in Development, Disease, and Therapeutic Target

**DOI:** 10.3390/cells14171391

**Published:** 2025-09-05

**Authors:** Abdulmajeed F. Alrefaei

**Affiliations:** Department of Biology/Genetic and Molecular Biology Central Laboratory (GMCL), Jamoum University College, Umm Al-Qura University, Makkah 2203, Saudi Arabia; afrefaei@uqu.edu.sa

**Keywords:** low density lipoprotein receptor-related protein-5, LRP5, Wnt signaling pathway, Wnt/β-catenin, Anti-DKK-1, anti-sclerostin, osteoporosis, lipid metabolism, fibrosis, cancer, angiogenesis

## Abstract

Low-density lipoprotein receptor-related protein 5 (LRP5) is a multifunctional transmembrane coreceptor that plays a pivotal role in development and disease. Wnt/β-catenin signaling is the primary downstream signaling pathway activated by LRP5. Furthermore, some LRP5 functions are mediated by noncanonical pathways, such as AKT/P21 and TGF-β/Smad signaling. Pathologically, both loss-of-function and gain-of-function mutations in LRP5 produce distinct phenotypes, ranging from osteoporosis-pseudoglioma syndrome to high bone mass disorders. Beyond the skeletal system, LRP5 has emerged as a key regulator of retinal angiogenesis, vascular integrity, renal tubular function, neurodevelopment, and lipid metabolism. Its physiological functions are highlighted by its ability to influence adipocyte differentiation, insulin sensitivity, and neuronal synaptic plasticity. Moreover, LRP5 displays a dual role in development and disease progression. Although it plays a protective role in acute injuries such as myocardial infarction and acute kidney injury, LRP5 also contributes to chronic pathologies such as tubulointerstitial fibrosis, polycystic kidney disease, and atherosclerosis through fibrotic and inflammatory pathways. Recent therapeutic interest has focused on modulating LRP5 activity using agents such as anti-Dickkopf-related protein 1 antibody, sclerostin inhibitors, polyclonal antibodies, CRISPR/Cas9 knockout, and some natural products. This review discusses the current understanding of LRP5's physiological and pathological roles across organ systems and highlights its therapeutic potential, emphasizing the need for targeted approaches considering its context-dependent effects.

## 1. Introduction

The Wnt/β-catenin signaling pathway is an essential key regulator of human physiology and pathology, mediating cellular differentiation, tissue development, homeostasis, and cell injury and repair [1]. Low-density lipoprotein receptor-related protein 5 (LRP5) is a crucial mediator of the canonical Wnt/β-catenin pathway, acting as an essential coreceptor in Wnt signal transduction initiation. After binding to the transmembrane Frizzled (Fzd) receptor, the Wnt ligand attaches to LRP5, initiating a conformational change in LRP5 that results in axin recruitment. Subsequently, the β-catenin complex is dissociated by axin-mediated inhibition of glycogen synthase kinase-3 (GSK3) [2,3,4]. This dissociation stabilizes β-catenin by inhibiting its phosphorylation, and β-catenin subsequently translocates to the nucleus, where it mediates Wnt gene expression via T-cell factor and lymphoid enhancer-binding factor families [5].

LRP5 and LRP6 demonstrate 71% sequence homology and belong to a specialized group of low-density lipoprotein (LDL) receptor-related proteins [6,7,8]. Each coreceptor has four Tyr-Trp-Thr-Asp (YWTD)-β-propeller domains and epidermal growth factor (EGF)-like domains that bind extracellular ligands. LRP5/6 also possess five phospho-protein phosphatase serine/threonine protein (PPP(S/T) P) domains that are essential for downstream signaling transmission [9].

The LRP5 gene, located on chromosome 11q13.4, regulates embryogenesis, bone homeostasis, retinal vascularization, neurogenesis, and metabolic processes through Wnt signaling. Mutations in LRP5 lead to distinct phenotypic outcomes, ranging from osteoporosis to excessive bone formation, and contribute to congenital retinal disorders, neurodevelopmental defects, and cancers [10]. Thus, LRP5 is a potential therapeutic target in multiple diseases. Nie et al. demonstrated that knocking down LRP5 expression via the clustered, regularly interspaced, short palindromic repeats (CRISPR)/CRISPR-associated 9 (CRISPR/Cas9) system could inhibit cancer cell growth [11]. Similarly, Guo et al. reported that overexpression of dominant-negative, soluble LRP5 (sLRP5) blocks Wnt signaling and reverses the epithelial–mesenchymal transition in tumor cells [12]. Although numerous studies have described the role of LRP5 in various diseases [13,14,15,16,17,18], the dual role of LRP5 in normal human development and pathology remains underreported. 

In humans, LRP5 is expressed in numerous tissues and organs, including the liver, pancreas, skeletal muscle, kidney, heart, and lung. The brain and peripheral leukocytes demonstrate relatively low LRP5 expression levels. LRP5 is an essential coreceptor in multiple metabolic pathways and plays a paradoxical and context-dependent role in human development, physiology, and pathology. LRP5 is a critical regulator of development during embryogenesis and after birth across multiple systems, including the bone, retina, and central nervous system; it is also involved in adipose tissue development, glucose regulation, and fat metabolism [13,14,15,16,17]. Conversely, LRP5 expression dysregulation contributes to pathological states such as osteoporosis–pseudoglioma syndrome [18], familial exudative vitreoretinopathy [19], high bone mass disorders [20], atherosclerosis [21], fibrotic renal and kidney diseases [22], and oncological diseases [23]. Although most of LRP5’s functions are mediated through canonical Wnt/β-catenin signaling, some are also mediated by other pathways, such as AKT/P21, TGF-β/Smad, and angiopoietin-Tie 2 signaling [22,24,25]. 

Clearly, comprehensive evidence describing LRP5’s role as a potential therapeutic target in systemic diseases is lacking. Thus, I aimed to comprehensively evaluate the physiological roles of LRP5 and the pathogenic mechanisms underlying LRP5 dysfunction, highlighting its role in clinical therapeutics through targeted exploitation. The dual role of LRP5 in various systems is summarized below.

## 2. LRP5 in Bone Formation and Skeletal Homeostasis

LRP5 is essential for osteoblast differentiation and bone mineralization, functioning as a coreceptor with Fzd receptors to activate Wnt signaling. LRP5 and LRP6 mRNA levels increase by up to 53% and 40%, respectively, on day 14 of osteoblast differentiation [13]. 

The gain of function mutation in LRP5 results in increased bone density, whereas its loss of function mutation causes osteoporosis-pseudoglioma syndrome [26]. LRP5 knockout mice develop severe osteoporosis, resembling human osteoporosis–pseudoglioma syndrome (OPPG); this phenotype arises from the disruption of Wnt3a-mediated Axin-2 expression, reduced bone mineralization, and increased expression of chondrocyte differentiation markers (Collagen II, Aggrecan, Collagen X, and Sox9), leading to the diversion of the osteoblast lineage to the chondrocyte lineage [13]. Conversely, gain-of-function mutations in LRP5 result in high bone mass (HBM) phenotypes, where increased Wnt signaling leads to excessive bone formation. The G171V point mutation in LRP5 leads to the inhibition of Dkk-1-mediated Wnt signaling antagonism, resulting in elevated fibronectin and TGF-β1 expression. TGF-β1 stimulates osteoblasts, resulting in high bone density. Thus, individuals with LRP5 (G171V) mutations exhibit a special phenotype comprising a straightened angle of the jaw, torus mandibularis, torus palatines, and a bone mineral density approximately three standard deviations higher than that observed in those without the mutation [20]. 

Loss of LRP5-mediated osteoblast function can be compensated until 8 weeks after birth in mice with LRP5 gene disruption in osteoclasts; however, alterations in both trabecular and cortical bone architecture occur by 16 weeks. LRP5 disruption in osteoblasts reduces the trabecular bone volume by 37%, the trabecular number by 36%, the cortical tissue area by 20%, cortical tissue thickness by 10%, the polar moment of inertia by 34%, and the mineralizing surface per bone surface area by 61%. Furthermore, the bone formation rate is reduced and the mineralization time is increased, with no change in the number of osteoblasts or osteoclasts [13].

**Figure 1:**
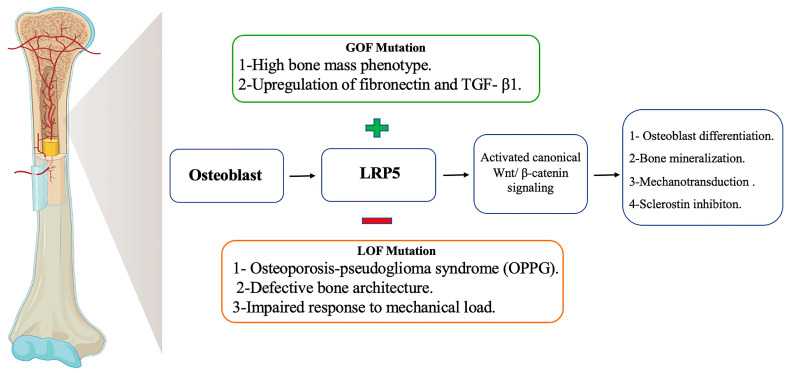
LRP5 functions and mutation effects in skeletal homeostasis.

LRP5 plays an important role in mechanotransduction—LRP5-deficient mice demonstrate a profound reduction (88–99%) in ulnar bone formation when subjected to mechanical loading [27]. However, LRP5 is not involved in PTH signaling-mediated bone homeostasis [27]. Mechanistically, LRP5 regulates sclerostin, a secreted protein that inhibits Wnt signaling and suppresses bone formation [28]. Therapeutically targeting sclerostin has shown efficacy in increasing bone mass [29]. Figure 1 highlights LRP5 functions and mutation effects in skeletal homeostasis.

Recently, exome sequencing analysis revealed that heterozygous mutations in the LRP5 and LGR4 genes had cumulative negative impacts on low bone mineral density, leading to bone deformities and recurring fractures. Additionally, these mutations extended their effects to extraskeletal sites, adversely affecting the patient's overall health [30]. 

## 3. LRP5 in Retinal Morphology and Ocular Diseases

LRP5 plays an important role in the development of retinal vasculature and the establishment of the blood–retinal barrier by acting as a coreceptor for Wnt signaling and other pathways. For example, LRP5 binds to the cell membrane Fzd4 receptor and participates in the Wnt-mediated nuclear translocation of β-catenin, leading to angiogenesis and the development of the retinal vascular–endothelial barrier. Loss-of-function mutations in LRP5 lead to defective vascularization, retinal detachment, and progressive vision loss [31,32]. LRP5 knockout rats demonstrate sparse retinal vasculature with lesser branching patterns, absent intermediate and deep vessels, and multiple autofluorescent exudates with avascular areas in the peripheral retina [33]. Furthermore, these rats demonstrate either a deficient outer nuclear layer or an outer nuclear layer merged with the inner nuclear layer, a lack of well-defined photoreceptors, and reduced thickness of the inner nuclear (20.2 ± 2.9 mm in knockout rats versus 28.0 ± 4.9 mm in wild-type rats) and plexiform (18.4 ± 3.6 μm in knockout rats versus 46.5 ± 10.3 μm in wild-type rats) layers [33]. Additionally, fundoscopic images of knockout rats were found to be hazy due to the preretinal membrane. Optical coherence tomography of LRP5 knockout animals revealed increased permeability of the retinal–blood barrier, evidenced by a 3.6-times greater accumulation of Evans blue dye in comparison with that observed in wild-type rats. In addition to structural changes, LRP5-deficient retinae are unresponsive to light per electroretinography [33]. 

**Figure 2:**
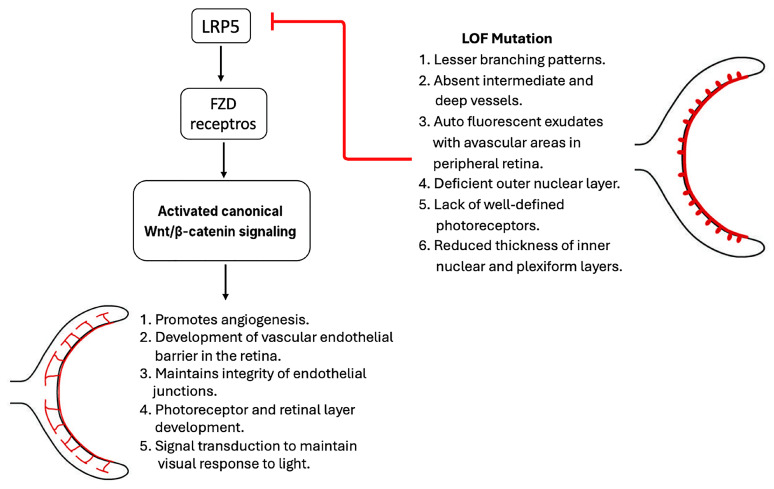
LRP5 in retinal development.

Individuals with homozygous inactivating mutations in LRP5, also known as OPPG, exhibit microphthalmia and impaired vision at birth due to poor retinal vascularization and pseudogliomas [18,34]. Similarly, in familial exudative vitreoretinopathy (FEVR), a hereditary disorder in humans characterized by inactivating mutations of LRP5, deficient peripheral retinal vascularization leads to progressive vision loss [35]. Hypoxia in the peripheral retina leads to neovascularization and posterior pole traction, resulting in retinal detachment. Additionally, the development of peripheral leaky vessels leads to exudate formation [19,36]. Several LRP5 mutations has been reported in premature babies with advanced retinopathy [37]. A recent study identified a novel pathogenic mutation in LRP5 in cases of FEVR [38]. A girl with anisometropic amblyopia showed signs of macular dragging and an avascular peripheral retina in her right eye. Whole exon sequencing revealed a novel heterozygous missense variant in the LRP5 gene, Glu528Lys, that is pathogenic. At the same time, her mother also possessed the same mutation, demonstrating peripheral exudations, areas of avascularity, and several microaneurysms. Interestingly, both cases showed unique phenotypes of familial exudative vitreoretinopathy. Figure 2 shows the important functions of LRP5 in retinal development and the phenotypes of its loss mutations.

## 4. LRP5 in the Cardiovascular and Pulmonary Systems

LRP5 variants have been linked to coronary artery disease (CAD), atherosclerosis, and insulin resistance [21,39,40]. The role of LRP5 in endothelial function and lipid metabolism suggests the potential for targeting this protein in metabolic and cardiovascular disease interventions [13].

LRP5 shortens the QT interval by modulating the proteasomal degradation of CaV1.2α1c protein, an essential component of L-type calcium channels in ventricular myocytes [41]. The QT interval is reduced by ~25% in LRP5 knockout mice through shortening of the APD at 90° repolarization, occurring independently of the Wnt signaling pathway; however, no effect on heart function and structure was observed, as the ejection fraction, cardiomyocyte size, and wall thickness were normal [41]. LRP5 levels are also increased in post-MI cardiac myocytes in response to cell injury [39]. LRP5 plays a protective role in the vascular system by optimizing serum cholesterol levels via glucose homeostasis and chylomicron remnant hepatic clearance [16,40]. Additionally, LRP5 mediates triacylglycerol hydrolysis by interacting with VLDL in the liver, preventing fat deposition [16,40]. Mice lacking LRP5 exhibit higher serum cholesterol levels than their control counterparts when fed a high-fat diet, also demonstrating large atheromatous lesions [40]. However, LRP5 plays a dual role in lipid metabolism, whereby LRP5 expression upregulation after a surge in fat intake activates the Wnt signaling pathway and promotes plaque formation through BMP2 and osteopontin (OPN) activation and the conversion of macrophages to foam cells [21]. The paradoxical role of LRP5 in atherosclerosis warrants further investigation.

**Figure 3:**
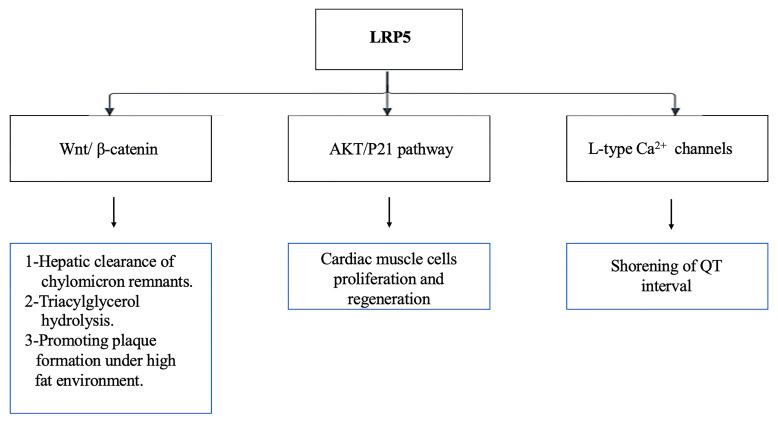
Role of LRP5 in the cardiovascular system.

LRP5 also plays a critical role in cardiac muscle cell proliferation and postnatal regeneration. LRP5 expression decreases with increasing age, resulting in the transition of cardiac cells from into permanent cells. The action is mediated by the AKT/P21 pathway, rather than Wnt signaling. LRP5 modulates the proteasomal degradation of AKT. Consequently, cellular markers of proliferation, such as EdU (DNA synthesis marker) and pH3-S10 (G2/mitosis marker), have been found to be reduced in LRP5-deficient mice [24]. Figure 3 exhibits the different roles of LRP5 in the cardiovascular system via diverse signaling pathways. 

LRP5 plays an important role in pulmonary vascular smooth muscle differentiation and proliferation via the activation of Cyclin D1 and WISP-1 downstream of the Wnt/β-catenin signaling pathway [25]. LRP5 expression is increased in pulmonary fibrosis and hypertension due to Wnt signaling pathway activation in response to hypoxia. Furthermore, LRP5 regulates the lung microvasculature via angiopoietin-Tie 2 pathway modulation, indicating that LRP5 is a potentially effective therapeutic target in pulmonary hypertension [42].

## 5. LRP5 in Renal Physiology and Pathology

LRP5 is abundant in the renal tubules of humans and mice, where it plays a dual role, protecting against diseases such as acute kidney injury but exerting a detrimental effect in other conditions such as tubulointerstitial fibrosis and polycystic kidney diseases [43]. LRP5-deficient mouse models demonstrate more extensive ischemia–reperfusion injury following acute kidney injury, with defective tissue repair and excessive renal tubule cystic transformation [43]. LRP5, via its profibrotic action, determines the prognosis of chronic kidney disease in both diabetic and non-diabetic models [22]. Tubulointerstitial fibrosis can be prevented in LRP5 knockout mice by inhibiting TGF-β/Smad signaling. LRP5 binds to TβRI and TβRII, leading to the formation of heterodimers with prolonged half-lives, promoting internalization into basal cells [22]. 

LRP5 is also involved in glomerulosclerosis and podocyte injury initiation in glomerular diseases via B7-1 and Hsp90ab1–β-catenin pathway interactions [44]. Some LRP5 variants also contribute to the development of adult polycystic kidney disease by reducing Wnt signaling pathway activation, as demonstrated by Luciferase assays [45]. In lupus nephritis, LRP5 mediates renal injury by modulating mitochondrial function and increasing apoptosis in HK-2 cells, acting synergistically with BPI fold-containing family A member 2 (BPIFA2), which is a parotid secretory protein and a biomarker of renal injury [46]. 

In addition to its pathogenic role in the renal system, LRP5 has recently been demonstrated to be renoprotective in renal tubular epithelial cells under lipid stress in diabetic kidney diseases [47]. RNA sequencing analysis of LRP5 knockout kidneys revealed reduced fatty acid oxidation mediated by the peroxisome proliferator-activated receptor (PPAR) signaling pathway. LRP5 expression downregulation affects epithelial cells' identity in renal tubules, exposing them to lipotoxic stress. LRP6 overexpression reduces the deposition of fatty acid intermediates and stimulates fatty acid oxidation, preventing lipotoxic stress in renal tubular epithelial cells. This structural and metabolic role of LRP5 presents it as a potential therapeutic target for kidney diseases, especially in individuals with diabetes with permanent renal tubular damage [47]. Additionally, this contradictory role of LRP5 in renal pathology necessitates future research to identify any cell-specific or environment-based variations in the profibrotic effect of LRP5.

## 6. LRP5 in Nervous System Development and Diseases

LRP5 has been observed in the nervous system of both animals and humans, playing a distinct role in neurodevelopment mediated by the canonical Wnt-signaling pathway. Grünblatt et al. conducted a meta-analysis and proposed a significant association between LRP5 and altered brain maturation among females with attention deficit hyperactivity disorder, demonstrated by simulating an Ala1330Val amino acid change [48]. In another animal study, LRP5 was found to be involved in brain development regulation mediated by Wnt3a–Fzd receptor protein 1 binding [18]. 

Unlike its direct role in cholesterol metabolism in the cardiovascular and renal systems, LRP5 is not involved in the direct absorption of cholesterol in neuronal cells. However, in the presence of LRP5, nLDL stimulates the canonical pathway by promoting the transcription of prosurvival proteins ADAM10 and MMP7. Additionally, LRP5 is important in maintaining life and neural development [49].

Gene set enrichment analysis of Lrp5−/− mouse brains revealed decreased synthesis of fatty acids and vitamins required for neuronal development and synapse formation via the downregulation of genes involved in retinol and linoleic acid metabolism [50]. Similarly, linoleic acid improves memory and reflex maturation in mice [51]. LRP5 is also responsible for cranial neural crest cell migration and craniofacial morphogenesis [52]. In a study on the role of LRP5 in cerebellar development, LRP5/6 knockout mice were found to exhibit postnatal growth inhibition, defective cerebellar development due to aberrant lamination and foliation, and β-catenin-mediated ectopic tyrosine hydroxylase expression in Purkinje cells [53]. LRP4 and LRP6 are also involved in forebrain and spinal cord development, and double mutants of LRP4 and LRP5 show neural tube defects [54]. 

## 7. LRP5 in Fat and Glucose Metabolism

LRP5 is associated with metabolic and insulin sensitivity. It exhibits a dualistic nature, protecting against lipid accumulation and promoting glucose homeostasis under physiological conditions, while facilitating foam cell formation and atherosclerosis when upregulated in inflammatory states [14,22,40]. LRP5 is known to influence body metabolism by regulating adipose cell proliferation, insulin sensitivity, and body fat distribution [55]. LRP5 regulates regional adiposity and directs fat towards the lower body using adipocyte progenitor cells as effector cells, regardless of their role in skeletal metabolism. Additionally, LRP5 results in lower body glucose, lower fasting insulin levels, increased adipose tissue insulin sensitivity, and controlled ADIPOQ expression [55]. 

LRP5 plays a essential role in the regulation of lower-body fat distribution and insulin sensitivity. Loh et al. reported increased abdominal fat distribution and reduced gluteal fat mass after LRP5 knockdown in gluteal and abdominal adipocytes. LRP5 stimulates insulin secretion from beta cells by modulating the murine Wnt-3a-stimulated glucose pathway [56]. Loss-of-function mutations in LRP5 have been found to result in abnormal glucose tolerance and hypercholesterolemia after a high-fat diet in animal studies due to a reduction in the levels of mRNA encoding insulin receptor, glucokinase, and HNF-4 [16]. Similarly, insulin resistance has been observed in humans with diminished white adipose tissue LRP5 expression [17]. A new study reported that variations at the LRP5-rs4988331 locus, along with the LRP6 variants, could be linked to the onset of Postmenopausal Type 2 Diabetes and Obesity [57].

## 8. LRP5 in Oncogenesis

LRP5 dysregulation is known to increase tumor cell proliferation in various cancers [58,59,60]. In squamous cell carcinoma of the tongue, LRP5 knockdown has been found to increase MMP1 in the CAL27 and SCC25 cell lines; furthermore, it increased PCNA levels, decreased apoptotic protein cleaved caspase-3 levels, and decreased adhesion-related proteins E-cad and β-catenin levels [23]. Collectively, these effects result in decreased tumor growth and proliferation through compensatory activation of the Akt pathway [23]. 

LRP5 gene fusion with UBE3C results in the loss of the DKK-1 inhibitory domain; in a study of head and neck cancers, this fusion was found to stimulate Wnt/β-catenin signaling and upregulate MYC, CCND1, TCF4, and LEF13 expression, resulting in tumor cell proliferation [58].

In breast carcinoma, the LRP5Δ receptor (internally truncated LRP5 receptor) is expressed at all disease stages and is involved in tumor cell proliferation and growth through Wnt3-mediated β-catenin-driven transcription activation without DKK-1 [59]. This finding suggests that LRP5Δ is a potential therapeutic target in breast carcinomas [59]. Moreover, LRP5 is overexpressed in and regulates tumor growth in triple-negative breast cancer by regulating STK-40 expression, another potential therapeutic target in breast cancer [60]. Consistent with this finding, LRP5 knockdown induced cell death (apoptosis) to a greater extent than did LRP6 knockdown.

LRP5 expression upregulation has been observed in various cancer types, including gastric cancer, prostate cancer, and osteosarcoma. The cancer-promoting properties of LRP5 are primarily Wnt signaling activation and alteration of the ATP supply through aerobic glycolysis [61,62,63,64,65]. Feng et al. reported increased LRP5 expression in gliomas and suggested that LRP5 is responsible for increased cell proliferation in this cancer type through MAPK/P53/CDC2 signaling regulation [66]. In prostate cancer, rtSPIRE1 stabilizes LRP5 and activates the PI3K/AKT signaling pathway to enhance cell proliferation and migration [67].

In gastric cancer, SNX5 prevents the internalization of LRP5 and enhances its recycling back to the cell membrane, thereby averting its degradation in the lysosome [68]. This increases the amount of LRP5 on the cell membrane, which in turn activates the Wnt/β-catenin signaling pathway. The activation results the development and advancement of this cancer type.

## 9. LRP5 as a Pharmacological Target

Given the pleotropic functionality of LRP5, it has been proposed as a potential therapeutic target in certain diseases. Anti-LRP5 polyclonal antibodies are widely used as antitumor drugs in breast carcinoma, reducing tumor cell viability and enhancing apoptosis by reducing β-catenin activity [55]. In head and neck cancers, pyrvinium pamoate has been shown to reduce UBE3C–LRP fusion gene-expressing tumor cell transformation via the degradation of β-catenin in a dose-dependent manner [54]. In addition, two drugs were identified via bioinformatic tools (Parthenolide and Vorinostat) and repurposed to target LRP5 expression in some cancer cell lines. Both drugs have induced some inhibitory effects on LRP5 expression [69]. In gastric cancer, researchers have targeted LRP5 using the CRISPR/Cas9 knockout system, which led to a reduction in the proliferation of cancer cells in vitro and in vivo via cell cycle-associated gene inhibition [11]. Recently, bioinformatic analysis identified LRP5 as a prognostic marker in glioblastoma. Daidzin (natural product) reduced LRP5 activity, lowered p-GSK-3β levels, and facilitated the degradation of c-Myc, effectively inhibiting the Wnt signaling pathway [70]. These results emphasize LRP5 as a potential target for therapy, with Daidzin proving to be an effective inhibitor of LRP5 and demonstrating considerable antitumor activity in glioblastoma.

The action of LRP5 on bone is mediated via DKK-1 antagonism of the Wnt pathway; hence, in osteoporosis, several antibodies targeting DKK-1 have been designed and tested in animal models, demonstrating excellent results. Anti-DKK1, BHQ88,0, DKK1-specific IgGs, and RH2-18 have been found to increase bone formation and decrease osteolytic lesions [71,72,73,74]. Despite the noted effectiveness of DKK-1 inhibitors in osteoporosis, concerns have been raised regarding the increased risk of malignancies due to increased Wnt signaling pathway activation; thus, their safety and efficacy in humans remain to be established [75]. In addition to its mediation activity in the skeletal system, LRP5 plays a role in skeletal homeostasis, which is mediated by intestinal serotonin; furthermore, the use of tryptophan hydroxylase as a therapeutic option is contradictory and requires further research [76,77,78]. Recombinant sclerostin inhibits the interaction of LRP5/6 with Fzd receptors, inhibiting Wnt signaling pathway activation [79]. Some potential therapeutic options targeting LRP5–sclerostin binding have also been found to reduce the risk of fractures. Romozosumab, blosozumab, and BPS804 are potential anti-sclerostin agents that have been tested in various clinical trials with satisfactory results, although allergic reactions occurred [80,81,82]. Recently, semaglutide, a GLP1 receptor agonist frequently used in diabetes, has shown promising effects in the osteogenic differentiation of bone marrow stem cells via Wnt/LRP5/β-catenin signaling pathway activation in in vitro studies, and there is a need for in vivo studies to confirm this effect [83]. 

In periodontitis, down-regulation of LRP5 results in the loss of alveolar bone and increases inflammation through the blockade of the PI3K/c-FOS signaling pathway, suggesting that LRP5 could be a promising target therapy for this disease [84]. Ang1–LRP5–Tie2 pathway activation by platelet-rich plasma protein is effective in alveolar regeneration after pneumonectomy, indicating its potential in the treatment of chronic lung diseases and lung regeneration after pneumonectomy [42]. 

In myocardial infarction, targeting LRP5 restores the viability of myocardial cells and promotes healing after hypoxia by modulating the canonical Wnt pathway [39]. In atherosclerosis, targeted inhibition of LRP5 in plaque-resident macrophages alters lipid uptake and presents a novel therapeutic avenue to mitigate lesion progression while potentially preserving its systemic metabolic benefits [21]. 

SZN-413, a FZD4/LRP5-targeting Norrin mimetic molecule, is an essential therapeutic option for diabetic retinopathy [85]. SZN-413 stimulates tight junction proteins (claudin-5 and zonula occludens-1) in retinal vasculature endothelial cells, reducing neovascular tufts, VEGF-induced pathologic leakage from the neovasculature, and areas of retinal ischemia [85]. Similarly, F4L5.13, a tetravalent diabody with a dumbbell-like structure, has also been found to demonstrate comparable results in the treatment of diabetic retinopathy by acting against FZD4 and LRP5 receptors and LRP5 signaling [86]. 

Furthermore, plant dietary supplements such as plant sterol esters (PSE) could reduce plasma lipid levels and lower plasma cholesterol. Such supplements abolish hypercholesterolemia diet-induced LRP5 expression levels and decreases atherosclerotic plaque coverage in the aorta [40]. The potential therapeutic targets of LRP5 are provided below in Table 1. 

## 10. Potential Challenges and Future Directions

Despite current advancements in targeting LRP5, several limitations and potential challenges remain. LRP5 regulates virous range of important biological activities beyond bone formation, including metabolism, cell proliferation, eye vascularization, and embryonic development. LRP5 dysfunction is reported in multiple organs such as its loss-of-function mutations are linked to both low bone mass and a rare congenital eye disease (Figure 1 and Figure 2). Thus, some present drugs that target LRP5 could have low specificity and off-target effects, potentially resulting in negative sides effects in other tissues and organs. For example, a therapeutic approach that targets bone diseases might lead to negative or even adverse effects on other systems. Another challenge is that Wnt pathway is complex because it is involved several ligands, 10 receptors and co-receptors including LRP5 [4,8]. In addition, some Wnt co-receptor have redundant functions such as LRP6 so targeting LRP5 alone might not be sufficient to reach the desired therapeutic outcome and reduce the efficacy of this approach. Furthermore, Wnt pathway activation to increase bone formation has been associated with the risk of cancer, raising concerns about the safety of LRP5 activation therapy. LRP5 also interacts with other signaling pathways and mediates diverse biological functions independent from the Wnt pathway. Therefore, developers of LRP5-based therapies should consider these complexities to develop specific therapeutical approaches. 

It is important to mention that variations in the LRP5 gene show diverse phenotypes. For example, A group of seven children (five males) possesses various variants in the LRP5 gene. Eight heterozygous variants (two nonsense and six missense) of the LRP5 gene were discovered, with two being potentially pathogenic [87]. In a computational analysis of 17 recognized nonsynonymous SNPs in the LRP5 gene, it was found that 14 of these variants caused damage at highly conserved locations, leading to the destabilization of both the protein's structure and function [88]. These variants underscore the different phenotypes associated with variations in the LRP5 gene, indicating a need for additional investigation on targeted precision therapies that affect Wnt signaling, and other pathways influenced by alterations in this gene.

Future research should focus on the development of novel drug delivery for LRP5-related bone diseases, included small molecules, localized implants, and targeted nanoparticles. These approaches would maximize the therapeutic dose and reduce off-target effects. LRP5-based therapies require more investigation into their long-term safety and dosing through biological experiments and clinical trials. Additionally, developing reliable biomarkers for LRP5 related diseases is essential to improve diagnosis and identify which patients will benefit from LRP5-targeted therapies. This could be achieved through genetic screening and other medical measurements. Ultimately, this will lead to the development of precision medicine strategies for LRP5 related conditions depend on the genetic variations in this gene, because one approach for all patients will not be efficient.

## 11. Conclusions

LRP5 plays a critical role in the human body, acting both as a guardian of development and as a driver of diseases in various systems. Although LRP5 plays pivotal roles in glucose and fat metabolism and skeletal, retinal, and neural development, loss-of-function mutations lead to systemic diseases such as OPPS, impaired glucose tolerance, and disrupted blood–retinal barrier integrity. Additionally, gain-of-function mutations result in high bone mass phenotypes, vascular calcification, and proliferative responses in specific tissues. The dual role of LRP5 is more evident in the cardiovascular and renal systems, where evidence regarding both protective and pathological roles is available. The key findings from this review should be utilized to clarify the complex signaling pathways downstream of LRP5 to understand its protective versus pathological roles in a disease-specific context. It will be essential to investigate how interactions with other receptors and pathways regulate LRP5 function. In addition, leveraging CRISPR-based gene editing and RNA-based therapy technologies could provide unprecedented insights for the development of LRP5-targeted therapy.

## Figures and Tables

**Table 1:** The summary of potential therapeutic targets of LRP5.

Therapeutic Agent/Tools	Stage of Development	Mechanism	System/Disease	Action	Study
CRISPR/Cas9 knockout	Preclinic	Downregulate LRP5 activity	Gastric cancer	Reduces the proliferation of cancer cells in vitro and in vivo by inhibiting the cell cycle-associated genes	[11]
Anti-LRP5 polyclonal antibody	Preclinic	Prevents LRP5 from interacting with key Wnt ligands and coreceptors, leading to attenuation of β-catenin activity	Breast cancer	Reduces tumor growth	[59]
Daidzin	Preclinic	Inhibitor of LRP5	glioblastoma	Reduced LRP5 activity, lowered p-GSK-3β levels, and facilitated the degradation of c-Myc	[70]
BHQ880 (anti-Dkk1 antibody)	Clinic	Activation of Wnt signaling by inhibiting DKK-1 antagonism of LRP5/β-catenin pathway	Osteoporosis in myeloma	Stimulates bone formation and inhibits bone resorption	[71,72]
RH2-18 (monoclonal anti-DKK1 antibody)	Preclinic	Activation of Wnt signaling by inhibiting DKK-1 antagonism of LRP5/β-catenin pathway	Osteoporosis	Promotes bone mineralization and rebuilds the structure of spongy bone	[74]
Blosozumab	Clinic	Binds specifically to sclerostin, preventing it from interacting with LRP5/6	Osteoporosis	Stimulates bone formation and decreases bone resorption	[80]
Romozosumab	Clinic	Binds specifically to sclerostin, preventing it from interacting with LRP5/6, Wnt pathway is subsequently activated	Osteoporosis	Stimulates bone mineralization and reduces bone resorption	[81]
BPS804 (anti-sclerostin antibody)	Clinic	binds specifically to sclerostin, preventing it from interacting with LRP5/6	Osteoporosis	Stimulates bone formation and inhibits bone resorption	[82]
Semaglutide	Preclinic	Increased activation of Wnt/LRP5/β-catenin signaling	Osteoporosis	Osteogenic differentiation of bone marrow stem cells	[83]
Pyrvinium pamoate	Preclinic	Could inhibit LRP5/Wnt/ β-catenin signaling	Head and neck cancers	Reduced tumor cell proliferation	[58]
Platelet-rich plasma extraction	Preclinic	Activation of Ang1–LRP5–Tie2 pathway	Chronic lung disease	Accelerates lung regeneration	[42]
Plant sterol esters	Preclinic	Prevent high cholesterol-induced LRP5 overexpression	Atherosclerosis	Downregulate pro-atherogenic genes and reduce vascular inflammation	[40]
SZN-413	Preclinic	FZD4/LRP5 agonist	Diabetic retinopathy	Angiogenesis and tightening of the blood–retinal barrier	[85]
F4L5.13	Preclinic	FZD4/LRP5 agonist	Diabetic retinopathy	Improves the integrity of retinal capillaries	[86]
Parthenolide and vorinostat	Preclinic	Downregulate LRP5 expression	Cancer	Possibly reduce cancer invasion and proliferation	[69]

## Data Availability

Not applicable.

## Abbreviations

ATP	Adenosine Triphosphate
ADAM	A Disintegrin And Metalloprotease
BFR	Bone Formation Rate
BPIFA2	BPI Fold-Containing Family A Member 2
BS	Bone Survival
CAD	Coronary Artery Disease
DKK-1	Dickkopf-1
FEVR	Familial Exudative Vitreoretinopathy
Fzd receptor	Frizzled Receptor
GSK-3	Glycogen Synthase Kinase-3
HBM	High Bone Mass
HNF-4	Hepatocyte Nuclear Factor 4
Hsp90	Heat Shock Protein 90
LRP5	Low-Density Lipoprotein Receptor-Related Protein 5
MMP-7	Matrix Metalloproteinase-7
mRNA	Messenger Ribonucleic Acid
OPPG	Osteoporosis-Pseudoglioma Syndrome
OPN	Osteopontin
PCNA	Proliferating Cell Nuclear Antigen
PGE2	Prostaglandin-E2
PPAR	Peroxisome Proliferator-Activated Receptor
sLRP5	Soluble Low-Density Lipoprotein Receptor-Related Protein 5
TGF	Transforming Growth Factor
VLDL	Very-Low-Density Lipoprotein
WISP-1	WNT1-Inducible-Signaling Pathway Protein 1

## References

[1] Maurice M.M., Angers S. (2025). Mechanistic insights into Wnt-β-catenin pathway activation and signal transduction. Nat. Rev. Mol. Cell Biol..

[2] Nusse R., Clevers H. (2017). Wnt/β-Catenin Signaling, Disease, and Emerging Therapeutic Modalities. Cell.

[3] Alrefaei A.F., Münsterberg A.E., Wheeler G.N. (2020). FZD10 regulates cell proliferation and mediates Wnt1 induced neurogenesis in the developing spinal cord. PLoS ONE.

[4] Alrefaei A.F. (2021). Frizzled receptors (FZD) play multiple cellular roles in development, in diseases, and as potential therapeutic targets. J. King Saud Univ. Sci..

[5] Rao T.P., Kühl M. (2010). An updated overview on Wnt signaling pathways: A prelude for more. Circ. Res..

[6] MacDonald B.T., He X. (2012). Frizzled and LRP5/6 receptors for Wnt/β-catenin signaling. Cold Spring Harb. Perspect. Biol..

[7] He X., Semenov M., Tamai K., Zeng X. (2004). LDL receptor-related proteins 5 and 6 in Wnt/beta-catenin signaling: Arrows point the way. Dev. Camb. Engl..

[8] Alrefaei A.F., Abu-Elmagd M. (2022). LRP6 Receptor Plays Essential Functions in Development and Human Diseases. Genes.

[9] Ren Q., Chen J., Liu Y. (2021). LRP5 and LRP6 in Wnt Signaling: Similarity and Divergence. Front. Cell Dev. Biol..

[10] Joiner D.M., Ke J., Zhong Z., Xu H.E., Williams B.O. (2013). LRP5 and LRP6 in development and disease. Trends Endocrinol. Metab. TEM.

[11] Nie X., Wang H., Wei X., Li L., Xue T., Fan L., Ma H., Xia Y., Wang Y.-D., Chen W.-D. (2022). LRP5 Promotes Gastric Cancer via Activating Canonical Wnt/β-Catenin and Glycolysis Pathways. Am. J. Pathol..

[12] Guo Y., Zi X., Koontz Z., Kim A., Xie J., Gorlick R., Holcombe R.F., Hoang B.H. (2007). Blocking Wnt/LRP5 signaling by a soluble receptor modulates the epithelial to mesenchymal transition and suppresses met and metalloproteinases in osteosarcoma Saos-2 cells. J. Orthop. Res. Off. Publ. Orthop. Res. Soc..

[13] Riddle R.C., Diegel C.R., Leslie J.M., Van Koevering K.K., Faugere M.-C., Clemens T.L., Williams B.O. (2013). Lrp5 and Lrp6 exert overlapping functions in osteoblasts during postnatal bone acquisition. PLoS ONE.

[14] Zhou Y., Wang Y., Tischfield M., Williams J., Smallwood P.M., Rattner A., Taketo M.M., Nathans J. (2014). Canonical WNT signaling components in vascular development and barrier formation. J. Clin. Investig..

[15] Veerapathiran S., Teh C., Zhu S., Kartigayen I., Korzh V., Matsudaira P.T., Wohland T. (2020). Wnt3 distribution in the zebrafish brain is determined by expression, diffusion and multiple molecular interactions. eLife.

[16] Fujino T., Asaba H., Kang M.-J., Ikeda Y., Sone H., Takada S., Kim D.-H., Ioka R.X., Ono M., Tomoyori H. (2003). Low-density lipoprotein receptor-related protein 5 (LRP5) is essential for normal cholesterol metabolism and glucose-induced insulin secretion. Proc. Natl. Acad. Sci. USA.

[17] Karczewska-Kupczewska M., Stefanowicz M., Matulewicz N., Nikołajuk A., Strączkowski M. (2016). Wnt Signaling Genes in Adipose Tissue and Skeletal Muscle of Humans With Different Degrees of Insulin Sensitivity. J. Clin. Endocrinol. Metab..

[18] Kandula A., Schenker K., Averill L. (2024). Osteoporosis-pseudoglioma syndrome. Pediatr. Radiol..

[19] Tao T., Xu N., Li J., Li H., Qu J., Yin H., Liang J., Zhao M., Li X., Huang L. (2021). Ocular Features and Mutation Spectrum of Patients With Familial Exudative Vitreoretinopathy. Investig. Ophthalmol. Vis. Sci..

[20] Boyden L.M., Mao J., Belsky J., Mitzner L., Farhi A., Mitnick M.A., Wu D., Insogna K., Lifton R.P. (2002). High bone density due to a mutation in LDL-receptor-related protein 5. N. Engl. J. Med..

[21] Borrell-Pagès M., Romero J.C., Juan-Babot O., Badimon L. (2011). Wnt pathway activation, cell migration, and lipid uptake is regulated by low-density lipoprotein receptor-related protein 5 in human macrophages. Eur. Heart J..

[22] He X., Cheng R., Huang C., Takahashi Y., Yang Y., Benyajati S., Chen Y., Zhang X.A., Ma J.-X. (2020). A novel role of LRP5 in tubulointerstitial fibrosis through activating TGF-β/Smad signaling. Signal Transduct. Target. Ther..

[23] Wang C., Li Y., Miao X., Wang Y., Yang G. (2024). Knockdown of LRP5 Promotes Proliferation and Invasion of Tongue Squamous Cell Carcinoma through Compensatory Activation of Akt Signaling. J. Cancer.

[24] Zhou H., Zhang F., Wu Y., Liu H., Duan R., Liu Y., Wang Y., He X., Zhang Y., Ma X. (2022). LRP5 regulates cardiomyocyte proliferation and neonatal heart regeneration by the AKT/P21 pathway. J. Cell Mol. Med..

[25] Alapati D., Rong M., Chen S., Lin C., Li Y., Wu S. (2013). Inhibition of LRP5/6-mediated Wnt/β-catenin signaling by Mesd attenuates hyperoxia-induced pulmonary hypertension in neonatal rats. Pediatr. Res..

[26] Ren N., Lv S., Li X., Shao C., Wang Z., Mei Y., Yang W., Fu W., Hu Y., Sha L. (2024). Clinical features, treatment, and follow-up of OPPG and high-bone-mass disorders: LRP5 is a key regulator of bone mass. Osteoporos. Int. J. Establ. Result Coop. Eur. Found. Osteoporos. Natl. Osteoporos. Found. USA.

[27] Sawakami K., Robling A.G., Ai M., Pitner N.D., Liu D., Warden S.J., Li J., Maye P., Rowe D.W., Duncan R.L. (2006). The Wnt co-receptor LRP5 is essential for skeletal mechanotransduction but not for the anabolic bone response to parathyroid hormone treatment. J. Biol. Chem..

[28] Maeda K., Kobayashi Y., Koide M., Uehara S., Okamoto M., Ishihara A., Kayama T., Saito M., Marumo K. (2019). The Regulation of Bone Metabolism and Disorders by Wnt Signaling. Int. J. Mol. Sci..

[29] Suen P.K., Qin L. (2016). Sclerostin, an emerging therapeutic target for treating osteoporosis and osteoporotic fracture: A general review. J. Orthop. Transl..

[30] Mehta P., Sharma A., Goswami A., Gupta S.K., Singhal V., Srivastava K.R., Chattopadhyay N., Singh R. (2024). Case report: Exome sequencing identified mutations in the LRP5 and LGR4 genes in a case of osteoporosis with recurrent fractures and extraskeletal manifestations. Front. Endocrinol..

[31] Chen J., Stahl A., Krah N.M., Seaward M.R., Dennison R.J., Sapieha P., Hua J., Hatton C.J., Juan A.M., Aderman C.M. (2011). Wnt signaling mediates pathological vascular growth in proliferative retinopathy. Circulation.

[32] Ubels J.L., Lin C.-M., Antonetti D.A., Diaz-Coranguez M., Diegel C.R., Williams B.O. (2022). Structure and function of the retina of low-density lipoprotein receptor-related protein 5 (Lrp5)-deficient rats. Exp. Eye Res..

[33] Ubels J.L., Diegel C.R., Foxa G.E., Ethen N.J., Lensing J.N., Madaj Z.B., Williams B.O. (2020). Low-Density Lipoprotein Receptor-Related Protein 5-Deficient Rats Have Reduced Bone Mass and Abnormal Development of the Retinal Vasculature. CRISPR J..

[34] Gowda V.K., Vegda H., Shivappa S.K., Benakappa N. (2020). Osteoporosis Pseudoglioma Syndrome. J. Pediatr. Neurosci..

[35] Cen C., He L., Hu K., Tao X., Liu Y., Li Q., Zhou W. (2025). An analysis of LRP5 gene frequencies in infants with familial exudative vitreoretinopathy in Chongqing and Urumqi. Medicine.

[36] Luvisi J.R., Blair K. (2025). Familial Exudative Vitreoretinopathy (FEVR). StatPearls [Internet].

[37] Kondo H., Kusaka S., Yoshinaga A., Uchio E., Tawara A., Tahira T. (2013). Genetic variants of FZD4 and LRP5 genes in patients with advanced retinopathy of prematurity. Mol. Vis..

[38] Chang H.-H., Wang A.-G., Niu D.-M., Chen Y.-R., Weng C.-C. (2024). Unveiling novel LRP5 pathogenic variant in familial exudative vitreoretinopathy: Diverse phenotypic expressions in a mother-daughter duo. Eur. J. Ophthalmol..

[39] Borrell M., Vilahur G., Romero J., Casani L., Badimon L. (2013). LRP5 modulates the Wnt signalling pathway to protect cardiac cells. Eur. Heart J..

[40] Borrell-Pages M., Romero J.C., Badimon L. (2014). Cholesterol modulates LRP5 expression in the vessel wall. Atherosclerosis.

[41] Liang D., Wu Y., Zhou L., Chen Y., Liu H., Xie D., Huang J., Zhang Y., Liu Y., Zhu W. (2019). LRP5 controls cardiac QT interval by modulating the metabolic homeostasis of L-type calcium channel. Int. J. Cardiol..

[42] Mammoto T., Chen Z., Jiang A., Jiang E., Ingber D.E., Mammoto A. (2016). Acceleration of Lung Regeneration by Platelet-Rich Plasma Extract through the Low-Density Lipoprotein Receptor-Related Protein 5-Tie2 Pathway. Am. J. Respir. Cell Mol. Biol..

[43] Kawakami T., Ren S., Duffield J.S. (2013). Wnt signalling in kidney diseases: Dual roles in renal injury and repair. J. Pathol..

[44] Li J., Niu J., Min W., Ai J., Lin X., Miao J., Zhou S., Liang Y., Chen S., Ren Q. (2022). B7-1 mediates podocyte injury and glomerulosclerosis through communication with Hsp90ab1-LRP5-β-catenin pathway. Cell Death Differ..

[45] Cnossen W.R., te Morsche R.H.M., Hoischen A., Gilissen C., Venselaar H., Mehdi S., Bergmann C., Losekoot M., Breuning M.H., Peters D.J.M. (2016). LRP5 variants may contribute to ADPKD. Eur. J. Hum. Genet. EJHG.

[46] Liu Y., Tian Y., Wang H., Li N., Zhuo R., Cui H., Miao X., Liu J., Liu Q., Zhang W. (2025). The BPIFA2-LRP5 axis orchestrates mitochondrial dysfunction to mediate kidney injury in lupus nephritis. Int. Immunopharmacol..

[47] HE X., ZENG R., LI H., WEN S., LI S., CHEN Y. (2025). 3-OR: LRP5 Promotes Fatty Acid Oxidation to Reduce Lipid Deposition in the Proximal Tubules of DKD Kidneys. Diabetes.

[48] Grünblatt E., Nemoda Z., Werling A.M., Roth A., Angyal N., Tarnok Z., Thomsen H., Peters T., Hinney A., Hebebrand J. (2019). The involvement of the canonical Wnt-signaling receptor LRP5 and LRP6 gene variants with ADHD and sexual dimorphism: Association study and meta-analysis. Am. J. Med. Genet. Part B Neuropsychiatr. Genet..

[49] Borrell-Pages M., Luquero A., Vilahur G., Padró T., Badimon L. (2024). Canonical Wnt pathway and the LDL receptor superfamily in neuronal cholesterol homeostasis and function. Cardiovasc. Res..

[50] Luquero A., Pimentel N., Vilahur G., Badimon L., Borrell-Pages M. (2024). Unique Splicing of Lrp5 in the Brain: A New Player in Neurodevelopment and Brain Maturation. Int. J. Mol. Sci..

[51] Queiroz M.P., da Lima M.S., Barbosa M.Q., de Melo M.F.F.T., de Bertozzo C.C.M.S., de Oliveira M.E.G., Bessa R.J.B., Alves S.P.A., Souza M.I.A., de do Queiroga R.C.R.E. (2019). Effect of Conjugated Linoleic Acid on Memory and Reflex Maturation in Rats Treated During Early Life. Front. Neurosci..

[52] Willems B., Tao S., Yu T., Huysseune A., Witten P.E., Winkler C. (2015). The Wnt Co-Receptor Lrp5 Is Required for Cranial Neural Crest Cell Migration in Zebrafish. PLoS ONE.

[53] Huang Y., Zhang Q., Song N.-N., Zhang L., Sun Y.-L., Hu L., Chen J.-Y., Zhu W., Li J., Ding Y.-Q. (2016). Lrp5/6 are required for cerebellar development and for suppressing TH expression in Purkinje cells via β-catenin. Mol. Brain.

[54] Geng S., Paul F., Kowalczyk I., Raimundo S., Sporbert A., Mamo T.M., Hammes A. (2023). Balancing WNT signalling in early forebrain development: The role of LRP4 as a modulator of LRP6 function. Front. Cell Dev. Biol..

[55] Loh N.Y., Vasan S.K., Rosoff D.B., Roberts E., van Dam A.D., Verma M., Phillips D., Wesolowska-Andersen A., Neville M.J., Noordam R. (2025). LRP5 promotes adipose progenitor cell fitness and adipocyte insulin sensitivity. Commun. Med..

[56] Loh N.Y., Neville M.J., Marinou K., Hardcastle S.A., Fielding B.A., Duncan E.L., McCarthy M.I., Tobias J.H., Gregson C.L., Karpe F. (2015). LRP5 regulates human body fat distribution by modulating adipose progenitor biology in a dose- and depot-specific fashion. Cell Metab..

[57] Li J., Li Y., Lu Y., Li S., Zhu Y., Sun C., Rai P., Jia X. (2025). The Relationship Between LRP-5 and LRP-6 Gene Mutations and Postmenopausal Type 2 Diabetes and Obesity. Clin. Med. Insights Endocrinol. Diabetes.

[58] Dharavath B., Butle A., Chaudhary A., Pal A., Desai S., Chowdhury A., Thorat R., Upadhyay P., Nair S., Dutt A. (2024). Recurrent UBE3C-LRP5 translocations in head and neck cancer with therapeutic implications. NPJ Precis. Oncol..

[59] Björklund P., Svedlund J., Olsson A.-K., Akerström G., Westin G. (2009). The internally truncated LRP5 receptor presents a therapeutic target in breast cancer. PLoS ONE.

[60] Maubant S., Tahtouh T., Brisson A., Maire V., Némati F., Tesson B., Ye M., Rigaill G., Noizet M., Dumont A. (2018). LRP5 regulates the expression of STK40, a new potential target in triple-negative breast cancers. Oncotarget.

[61] Zhang Y., Xia F., Liu X., Yu Z., Xie L., Liu L., Chen C., Jiang H., Hao X., He X. (2018). JAM3 maintains leukemia-initiating cell self-renewal through LRP5/AKT/β-catenin/CCND1 signaling. J. Clin. Investig..

[62] Björklund P., Akerström G., Westin G. (2007). An LRP5 receptor with internal deletion in hyperparathyroid tumors with implications for deregulated WNT/beta-catenin signaling. PLoS Med..

[63] Wang H., Deng G., Ai M., Xu Z., Mou T., Yu J., Liu H., Wang S., Li G. (2019). Hsp90ab1 stabilizes LRP5 to promote epithelial-mesenchymal transition via activating of AKT and Wnt/β-catenin signaling pathways in gastric cancer progression. Oncogene.

[64] Rabbani S.A., Arakelian A., Farookhi R. (2013). LRP5 knockdown: Effect on prostate cancer invasion growth and skeletal metastasis in vitro and in vivo. Cancer Med..

[65] Horne L., Avilucea F.R., Jin H., Barrott J.J., Smith-Fry K., Wang Y., Hoang B.H., Jones K.B. (2016). LRP5 Signaling in Osteosarcomagenesis: A Cautionary Tale of Translation from Cell Lines to Tumors. Transl. Oncol..

[66] Feng Y.-Y., Jin X., Pan M.-X., Liao J.-M., Huang X.-Z., Kang C.-M. (2025). LRP5 enhances glioma cell proliferation by modulating the MAPK/p53/cdc2 pathway. Int. J. Med. Sci..

[67] Hu J., Shi J., Wang J., Xiao Y., Kong D., Gao M., Luo T., Xu S., Yuan Z., Ma X. (2025). A novel polypeptide encoded by circSPIRE1 promotes prostate cancer proliferation and migration by restraining the ubiquitin-dependent degradation of LRP5. J. Exp. Clin. Cancer Res. CR.

[68] Le Y., Zhou L., He Y., Zhou J., Zhan J., Zhang H., Chen X., Xiong J., Fang Z., Xiang X. (2025). SNX5 facilitates the progression of gastric cancer by increasing the membrane localization of LRP5. Oncogene.

[69] Correa-Arzate L., Portilla-Robertson J., Ramírez-Jarquín J.O., Jacinto-Alemán L.F., Mejía-Velázquez C.P., Villanueva-Sánchez F.G., Rodríguez-Vázquez M. (2023). LRP5, SLC6A3, and SOX10 Expression in Conventional Ameloblastoma. Genes.

[70] Pan Y., Wang S., Duan G., Wu J., Feng F., Chen L., Li A., Xu K., Wang C., Fan S. (2025). Natural Product Daidzin Inhibits Glioma Development via Suppressing the LRP5-Mediated GSK-3β/c-Myc Signaling Pathway. BioFactors Oxf. Engl..

[71] Yaccoby S., Ling W., Zhan F., Walker R., Barlogie B., Shaughnessy J.D. (2007). Antibody-based inhibition of DKK1 suppresses tumor-induced bone resorption and multiple myeloma growth in vivo. Blood.

[72] Heath D.J., Chantry A.D., Buckle C.H., Coulton L., Shaughnessy J.D., Evans H.R., Snowden J.A., Stover D.R., Vanderkerken K., Croucher P.I. (2009). Inhibiting Dickkopf-1 (Dkk1) removes suppression of bone formation and prevents the development of osteolytic bone disease in multiple myeloma. J. Bone Miner. Res. Off. J. Am. Soc. Bone Miner. Res..

[73] Fulciniti M., Tassone P., Hideshima T., Vallet S., Nanjappa P., Ettenberg S.A., Shen Z., Patel N., Tai Y.-T., Chauhan D. (2009). Anti-DKK1 mAb (BHQ880) as a potential therapeutic agent for multiple myeloma. Blood.

[74] Glantschnig H., Scott K., Hampton R., Wei N., McCracken P., Nantermet P., Zhao J.Z., Vitelli S., Huang L., Haytko P. (2011). A rate-limiting role for Dickkopf-1 in bone formation and the remediation of bone loss in mouse and primate models of postmenopausal osteoporosis by an experimental therapeutic antibody. J. Pharmacol. Exp. Ther..

[75] Kim J.H., Liu X., Wang J., Chen X., Zhang H., Kim S.H., Cui J., Li R., Zhang W., Kong Y. (2013). Wnt signaling in bone formation and its therapeutic potential for bone diseases. Ther. Adv. Musculoskelet. Dis..

[76] Yadav V.K., Balaji S., Suresh P.S., Liu X.S., Lu X., Li Z., Guo X.E., Mann J.J., Balapure A.K., Gershon M.D. (2010). Pharmacological inhibition of gut-derived serotonin synthesis is a potential bone anabolic treatment for osteoporosis. Nat. Med..

[77] Inose H., Zhou B., Yadav V.K., Guo X.E., Karsenty G., Ducy P. (2011). Efficacy of serotonin inhibition in mouse models of bone loss. J. Bone Miner. Res. Off. J. Am. Soc. Bone Miner. Res..

[78] Cui Y., Niziolek P.J., MacDonald B.T., Zylstra C.R., Alenina N., Robinson D.R., Zhong Z., Matthes S., Jacobsen C.M., Conlon R.A. (2011). Lrp5 functions in bone to regulate bone mass. Nat. Med..

[79] Li X., Zhang Y., Kang H., Liu W., Liu P., Zhang J., Harris S.E., Wu D. (2005). Sclerostin binds to LRP5/6 and antagonizes canonical Wnt signaling. J. Biol. Chem..

[80] Su Y., Wang W., Liu F., Cai Y., Li N., Li H., Li G., Ma L. (2022). Blosozumab in the treatment of postmenopausal women with osteoporosis: A systematic review and meta-analysis. Ann. Palliat. Med..

[81] Shakeri A., Adanty C. (2020). Romosozumab (sclerostin monoclonal antibody) for the treatment of osteoporosis in postmenopausal women: A review. J. Popul. Ther. Clin. Pharmacol..

[82] Glorieux F.H., Devogelaer J.-P., Durigova M., Goemaere S., Hemsley S., Jakob F., Junker U., Ruckle J., Seefried L., Winkle P.J. (2017). BPS804 Anti-Sclerostin Antibody in Adults With Moderate Osteogenesis Imperfecta: Results of a Randomized Phase 2a Trial. J. Bone Miner. Res. Off. J. Am. Soc. Bone Miner. Res..

[83] Tian Y., Liu H., Bao X., Li Y. (2025). Semaglutide promotes the proliferation and osteogenic differentiation of bone-derived mesenchymal stem cells through activation of the Wnt/LRP5/β-catenin signaling pathway. Front. Pharmacol..

[84] Jiang H., Xi Y., Jiang Q., Dai W., Qin X., Zhang J., Jiang Z., Yang G., Chen Q. (2025). LRP5 Down-Regulation Exacerbates Inflam-mation and Alveolar Bone Loss in Periodontitis by Inhibiting PI3K/c-FOS Signalling. J. Clin. Periodontol..

[85] Nguyen H., Chen H., Vuppalapaty M., Whisler E., Logas K.R., Sampathkumar P., Fletcher R.B., Sura A., Suen N., Gupta S. (2022). SZN-413, a FZD4 Agonist, as a Potential Novel Therapeutic for the Treatment of Diabetic Retinopathy. Transl. Vis. Sci. Technol..

[86] Levey J., Douglas K., Jo H.-N., Zhang L., Seshagiri S., Angers S., Junge H.J. (2024). A Frizzled4/LRP5 agonist restores retinal pericyte coverage and reduces hemorrhage in a genetic model of pericyte loss. Investig. Ophthalmol. Vis. Sci..

[87] Ubertini G., Fintini D., d’Aniello F., Urbano F., Chiarito M., Angelelli A., Di Iorgi N., Faienza M.F. (2025). Clinical, Biochemical and Radiological Features of LRP5 Gene Variants in Children. Calcif. Tissue Int..

[88] Gorges D.M., Filippin-Monteiro F.B. (2025). Genetic variants in the LRP5 gene associated with gain and loss of bone mineral density. In Silico Pharmacol..

